# Genetic and Antimicrobial Resistance Profiles of Mammary Pathogenic *E. coli* (MPEC) Isolates from Bovine Clinical Mastitis

**DOI:** 10.3390/pathogens11121435

**Published:** 2022-11-28

**Authors:** Fernanda C. Campos, Ivana G. Castilho, Bruna F. Rossi, Érika C. R. Bonsaglia, Stéfani T. A. Dantas, Regiane C. B. Dias, Ary Fernandes Júnior, Rodrigo T. Hernandes, Carlos H. Camargo, Márcio G. Ribeiro, José C. F. Pantoja, Hélio Langoni, Vera L. M. Rall

**Affiliations:** 1 Department of Chemical and Biological Sciences, Institute of Biosciences, São Paulo State University, Botucatu 18618-691, Brazil; 2 Bacteriology Division, Adolfo Lutz Institute, São Paulo 01246-000, Brazil; 3 Department of Veterinary Hygiene and Public Health, School of Veterinary Medicine and Animal Sciences, São Paulo State University, Botucatu 18618-681, Brazil

**Keywords:** ESBL, phylogroup, intramammary infection, virulence, MPEC

## Abstract

Mammary pathogenic *E. coli* (MPEC) is one of the main pathogens of environmental origin responsible for causing clinical mastitis worldwide. Even though *E. coli* are strongly associated with transient or persistent mastitis and the economic impacts of this disease, the virulence factors involved in the pathogenesis of MPEC remain unknown. Our aim was to characterize 110 MPEC isolates obtained from the milk of cows with clinical mastitis, regarding the virulence factor-encoding genes present, adherence patterns on HeLa cells, and antimicrobial resistance profile. The MPEC isolates were classified mainly in phylogroups A (50.9%) and B1 (38.2%). None of the isolates harbored genes used for diarrheagenic *E. coli* classification, but 26 (23.6%) and 4 (3.6%) isolates produced the aggregative or diffuse adherence pattern, respectively. Among the 22 genes investigated, encoding virulence factors associated with extraintestinal pathogenic *E. coli* pathogenesis, *fimH* (93.6%) was the most frequent, followed by *traT* (77.3%) and *ompT* (68.2%). Pulsed-field gel electrophoresis analysis revealed six pulse-types with isolates obtained over time, thus indicating persistent intramammary infections. The genes encoding beta-lactamases detected were as follows: *bla_TEM_* (35/31.8%); *bla_CTX-M-2_*/*bla_CTX-M-8_* (2/1.8%); *bla_CTX-M-15_* and *bla_CMY-2_* (1/0.9%); five isolates were classified as extended spectrum beta-lactamase (ESBL) producers. As far as we know, *papA*, *shf*, *ireA*, *sat* and *bla_CTX-M-8_* were detected for the first time in MPEC. In summary, the genetic profile of the MPEC studied was highly heterogeneous, making it impossible to establish a common genetic profile useful for molecular MPEC classification. Moreover, the detection of ESBL-producing isolates is a serious public health concern.

## 1. Introduction

*Escherichia coli* is an opportunistic pathogen responsible for causing clinical bovine mastitis [1], causing losses (mainly lower milk value, treatment costs, and also the risk of culling) and affecting animal welfare.

In clinical bovine mastitis, the signals range from mild to severe, such as pain, fever, swelling, changes in rumination rate, hydration and behavior, and there can be animal death. Besides, there are changes in the milk, with the occurrence of flakes and clots, altered color and consistency [2]. In addition to *E. coli*, other environmental pathogens can also cause clinical mastitis, such as *Streptococcus uberis, Streptococcus dysgalactiae* [3] and non-aureus staphylococci [4]. Subclinical mastitis shows no systemic symptoms or visible changes in the milk, but there is an increase in somatic cell count (SCC) [5]. The main pathogens involved are *Staphylococcus aureus, Streptococcus agalactiae* and *Corynebacterium bovis* [3]. Considering the source of the agent, mastitis can also be classified as contagious, where the pathogen is mainly transmitted between animals and by milking equipment, or as environmental, caused by ubiquitous environmental pathogens, mostly found in bedding material, dust, flies and feces, such as *E. coli* [6].

On the basis of the virulence profile and distinct clinical outcomes observed in the host, pathogenic *E. coli* isolates are divided into diarrheagenic (DEC) or extraintestinal pathogenic *E. coli* (ExPEC); moreover, there are isolates that are harmless commensal microorganisms of the gastrointestinal tract [7]. Of importance, ExPEC isolates that cause clinical bovine mastitis are designated mammary pathogenic *E. coli* (MPEC) [8]. Phylogenetic analyses have revealed that *E. coli* exhibits a complex population substructure with the existence of eight distinct phylogroups, namely A, B1, B2, C, D, E, F, and G [9]. The majority of the MPEC isolates have been assigned to phylogroups A or B1 [10], which may suggest the environment as the principal source of this opportunistic pathogen.

Therapy using antimicrobial agents remains one of the main strategies for treatment of infectious diseases in dairy herds, including intramammary infections, especially cases of clinical mastitis [11].

Since specific virulence-encoding genes associated with the pathogenicity of *E. coli* causing bovine mastitis are still unknown, we investigated in the present study the virulence factor-encoding genes originally characterized in ExPEC isolates, Moreover, to better understand the genetic profile of these isolates, we investigated the presence of genes frequently used to classify *E. coli* in the distinct pathotypes of diarrheagenic *E. coli*.

## 2. Material and Methods

### 2.1. E. coli Isolates

We used 110 convenience samples of *E. coli* previously isolated from the milk of cows with clinical mastitis. These cases occurred on dairy farms located in the states of São Paulo, Minas Gerais and Paraná from 2014 to 2017, and the disease was diagnosed by macroscopic changes in the milk (using a strip cup) and/or physical symptoms in the animal (inflammation of the mammary gland and/or systemic signs in animals) [12]. Milk samples just from the quarter with clinical mastitis were collected in sterile tubes, after disinfection of the teats with 70% alcohol, and kept refrigerated until processing. This study was approved by the UNESP’s Animal Use Ethics Committee (N 0136/2017, approved in 6 June 2017). The milk samples were plated on MacConkey agar (MC, Oxoid) and defibrinated sheep blood agar (5.0%); the plates were incubated at 35 °C up to 72 h. Characteristic colonies were identified according to the National Mastitis Council [13]. After identification, the *E. coli* isolates were kept frozen at −70 °C in Brain Heart Infusion (BHI, Difco) broth with 20% glycerol, until analysis. Each isolate was plated on blood agar to verify the purity of the sample, before starting the tests.

### 2.2. Molecular Characterization of E. coli Isolates

For DNA extraction, from an overnight MC agar, one colony of each isolate was transferred to 200 µL of sterile Milli-Q water and boiled for 10 min, followed by centrifugation at 10,000 rpm for 1 min, and the supernatant was frozen for future PCR reactions [14].

*E. coli* isolates were classified into the different phylogenetic groups already recognized (A, B1, B2, C, D, E, F, G and *Escherichia* clades) according to Clermont et al. [15] and Clermont et al. [9]. The samples were tested in quadruplex PCR with primers for *arpA*, *chuA*, yjaA and *tspE4*.C2 genes, as recommended by Clermont et al. [15]. For confirmation of groups A or C in case of overlap, primers for *trpAgpC* were used for confirmation of group C and *trpBA* as an internal control of the reaction. For overlapping groups D and E, primers for *arpAgpE* and *trpBA* were used. For the quadruplex PCR, the standard strains 042 (*arpA*+ and *chuA*+) and E2348/69 (*chuA*+, *yjaA*+ and *tspE4C2*+) were used as positive controls. For confirmation of group C, *E. coli* EC51 (*trpAgpC*+) was the positive control and C600 was the negative, and for group E, *E. coli* EDL933 (*arpAgpE*+) and E2348/69 were the positive and negative controls, respectively. Using PCR reactions, the detection of virulence factor-encoding genes associated with DEC (*eae, bfpA, bfpB, aatA, aggR, eae, stx1, stx2, let, est,* and *ipaH*) was performed according to the references cited in Table S1 (Supplementary Materials).

Regarding ExPEC-related genes, we looked for genes related to: adhesins, i.e., *sfaDE, papC*, *afaBC* III, *fimA, ecpA*, *fimH, papA* and *iha*; toxins, i.e., *hlyA*, *cnf1*, *sat*, *vat* and *cdt*; siderophores, i.e., *iroN*, *irp2* I, *iucD*, *ireA* and *sitA*; invasins, i.e., *ibe10*; and serum resistance, i.e., *traT*, *KpsMTII* and *ompT* (primers and their characteristics and references are listed in Table S2).

The *astA* gene was investigated according to Savarino et al. [16]. Isolates that showed the aggregative adherence (AA) pattern and/or the *astA* gene were subjected to the investigation of genes commonly found in enteroaggregative *Escherichia coli* (EAEC), such as adhesins (*aggA*, *aafA, agg3A, agg4A, agg5A,* and *pilS*), toxins (*pic, sigA, sepA,* and *pet*) and miscellaneous (*shf, aap, aaiA, aaiC,* and *aaiG*) (primers and their characteristics and references are listed in Table S3).

Pulsed-field gel electrophoresis (PFGE) assay was performed according to PulseNet/CDC [17] protocols. The gels were stained with 1% ethidium bromide (Sigma- Aldrich, Missouri, MO, USA) for 30 min, followed by a 30-min wash with distilled water and photographed in a Major Science UVCI 1100 image analyzer (Saratoga, CA, USA). The images were analyzed using Bionumerics software v.7.6 (bioMérieux, Marcy-l’Étoile, France) with a tolerance of 1.5%. Clustering was carried out by the unweighted pair-group method with arithmetic mean (UPGMA), using the Dice coefficient. We used XbaI-digested DNA from ATCC BAA-664 (*Salmonella enterica* subsp. *enterica* serovar Braenderup H9812) as the molecular weight marker.

### 2.3. In Vitro Adhesion Test with HeLa Epithelial Cells

HeLa cells were always kept at 37 °C in an atmosphere of 5% CO_2_, in Dulbecco’s Modified Eagle Medium (DMEM) (Sigma), containing 10% fetal bovine serum (FBS) (Sigma) and 1% PenStrep antibiotic (10,000 U penicillin + 10 mg streptomycin/mL) (Sigma). The adhesion tests were performed according to Cravioto et al. [18]. Briefly, 1 mL of a suspension of HeLa cells at a concentration of 1 × 10^5^ cells/mL (Neubauer chamber) was distributed in a 24-well microplate containing glass coverslips, incubated at 37 °C in an atmosphere of 5% CO_2_ for 48 h, until reaching the semi-confluence stage (70 to 90%). A 20-µL aliquot of each isolate (cultured overnight in BHI broth at 35 °C, without agitation) was added in duplicate to the microplate wells, already containing HeLa cells in DMEM supplemented with 2% FBS and 2% methyl α-D-mannopyranoside (Sigma). After 6 h of incubation, each well was washed six times with sterile PBS, and the slides were fixed with methanol (overnight), stained with May–Grünwald (5 min) and Giemsa (20 min), and analyzed under an oil immersion microscope. Isolates EPEC-E2348/69 (AL standard), EAEC-042 (AA standard), DAEC-C1845 (AD standard) and *E. coli* HB101 (NA standard) were used as controls [19].

### 2.4. Antimicrobial Susceptibility Testing and Detection of the Resistance Genotypes

Antimicrobial susceptibility testing was performed according to the CLSI standards [19] using the disk diffusion technique, with the antimicrobials, ampicillin (AMP, 10 µg), cefepime (CPM, 30 µg), cefotaxime (CTX, 30 µg), ceftriaxone (CRO, 30 µg), cefoxitin (CFO, 30 µg), ceftazidime (CAZ, 30 µg), ceftiofur (CTF, 30 µg), aztreonam (ATM, 30 µg), gentamicin (GEN, 10 µg), streptomycin (EST, 10 µg) and tetracycline (ETT, 30 µg) (Cefar, São Paulo, Brazil).

The isolates were screened by the agar disk diffusion test, using ceftriaxone (CRO), cefotaxime (CTX), ceftazidime (CAZ) and aztreonam (ATM). Isolates with a halo smaller than or equal to that recommended by CLSI (2020) (CRO ≤ 25 mm, CTX ≤ 27 mm, CAZ ≤ 22 mm and ATM ≤ 27 mm) were selected for the confirmatory test, which consists of the same test with the addition of an amoxicillin-clavulanic acid disk (CAM, 30 µg) at a distance of 20 mm between the antibiotic disks, as a β-lactamase inhibitor. The presence of distorted halos or a “ghost zone” indicated ESBL-producing *E. coli* [20].

The 110 *E. coli* isolates were investigated for the presence of genes responsible for resistance including *bla_TEM_*, *bla_SHV_*, bla_CTX-M_, bla_CTX-M2_, bla_CTX-M8_, bla_CTX-M15_*bla_CMY2_*, *bla_KPC,_ bla_NDM_*, *mcr*-1 and *mcr*-2 (primers and their characteristics and references are listed in Table S4). Isolates that possessed the *bla_CTX-M_* gene were sequenced for classification of cefotaximase-Munich (CTX-M) by Sanger sequencing at the Institute of Biotechnology (Sao Paulo State University, UNESP).

## 3. Results

### 3.1. Molecular Features of the MPEC Isolates

The majority of the 110 isolates were assigned to *E. coli* phylogroups A (50.9%, 56/110) and B1 (38.2%, 42/110), despite the existence of MPEC in phylogroups D (2.7%, 3/110) and C (1.8%, 2/110). Less frequently, we observed one isolate (0.9%, 1/110) in each of the following groups: B2, E, F and E. clade I. Of note, three isolates (2.7%) were not classified in any of the *E. coli* phylogroups or *E.* clades identified so far.

Regarding the virulence genes associated with the pathogenicity of MPEC, *sfaDE, iha, afaBC* and *cnf1* were not observed. The frequency of the genes found, related to their respective phylogroups, are described in Table 1. One *E. coli* phylogroup B2 isolate possessed 12 (54.5%) of the 22 genes surveyed. Genes that confer the presence of adhesins, invasins and resistance to serum were found in all phylogroups, while siderophores were not present in groups C or E. clade I. The genes that encode toxins were the least frequent, absent in phylogroups D, E, F, E. clade I and in the unknown. The patterns of combinations between the 22 genes surveyed are given in Table S5.

**Figure 1:**
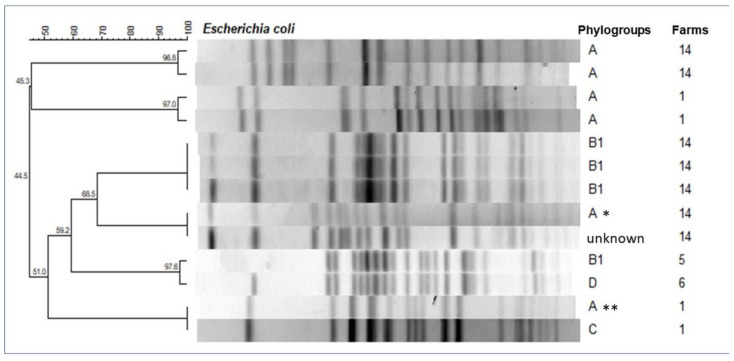
Dendrogram of PFGE patterns with >95% similarity coefficients for *E. coli* isolates obtained from the milk of cows with clinical mastitis. (*): 100% similarity between phylogroups A and unknown; (**), and between phylogroups A and C.

Through PFGE analysis, the chromosomal DNA of the isolates produced an average of 20 fragments in the range of 28.8 to 1135 Kb. In total, 103 pulsotypes were observed (Figure S1). Figure 1 shows the six (5.8%) clusters with >95% similarity and interestingly, one of these clusters (*) showed 100% similarity between two isolates from different phylogroups (A and unknown), as well as another (**), considering phylogroups A and C. These four isolates were resubmitted to PCR tests just to reconfirm their phylogroups.

Virulence factor-encoding genes frequently used for DEC pathotype identification were not detected in any of the MPEC isolates studied. However, 23.6% (26/110) produced AA and 3.6% (4/110) diffuse adherence (DA) patterns, which comprise important features of the aggregative (EAEC) and diffuse (DAEC) DEC pathotypes, respectively (Figure S2). It is also important to note that the *astA* gene, encoding an enteroaggregative *E. coli* heat-stable toxin, was detected in 7.3% (8/110) of the MPEC isolates studied, with 62.5% of them (5/8) also producing AA in HeLa cells. To better understand the genetic background of MPEC isolates harboring *astA* and/or producing AA in HeLa cells, we searched for a set of genes encoding virulence factors originally identified in the EAEC pathotype. The majority of the MPEC isolates AA^+^/*astA*^+^ lacked virulence genes from the EAEC pathotype, with *shf* (protein participating in cell–cell adhesion) being the only EAEC virulence factor-encoding gene detected in 3 AA-producing MPEC isolates lacking the *astA* gene (data not shown).

### 3.2. Antimicrobial Susceptibility Testing and Detection of the Resistance Genotypes

Resistance to at least one of the antimicrobial agents tested occurred in 29 (23.4%) isolates and nine (8.2%) showed multidrug resistance to at least three different groups of antimicrobials. Although the isolates showed moderate rates of resistance to tetracycline (19.1%) and ampicillin (12.7%), low rates were observed for cefoxitin (0.9%), gentamicin (3.6%) and ceftiofur (5.5%). The antibiogram results considering the isolates for each tested antimicrobial are given in Table S6.

Regarding in vitro screening tests for ESBL production, 42 (38.2%) isolates were resistant to aztreonam, 29 (26.4%) to cefotaxime, 12 (10.9%) to ceftazidime and 8 (7.3%) to ceftriaxone, totaling 47 (42.7%) isolates selected for the confirmatory test with the addition of the amoxicillin-clavulanic acid disk, and in 5 (4.5%), the formation of the “ghost zone” or distortion of the halos occurred, confirming ESBL production.

Considering the genetic resistance profile, the *bla_TEM_* gene was the most frequent, occurring in 35 (31.8%) isolates, while *bla_CTX-M-_*_2_/*bla_CTX-M-8_* and *bla_CTX-M-15_* were reported in two (1.8%) and one (0.9%) isolates, respectively. The positive isolates for *bla_CTX-M-2_* and *bla_CTX-M-15_* demonstrated the *bla_TEM_* gene concomitantly. The *bla_CMY-_*_2_ gene was reported in one (0.9%) isolate, along with the *bla_TEM_* gene. The *mcr*-1 and *mcr*-2 genes and the *bla_KPC_* and *bla_NDM_* genes that confer resistance to colistin and carbapenemases, respectively, were not found in any of the isolates.

## 4. Discussion

MPEC from the A and B1 phylogroups represented 89.1% of the total 110 isolates, which were also observed in high frequency in previous studies [10,21]. *E. coli* isolates assigned to these phylogroups are frequently found as commensal microorganisms in the gut or in the environment, and in this way, it is believed that MPEC infections can be better controlled with appropriate management practices (daily removal of waste and avoidance of moisture and environmental organic matter pre- and post-milking and pre-dipping, while offering food in the post-milking phase) to reduce the risk of contamination of the teats with feces that can accumulate on the farm and equipment facilities [22].

The pathogenicity of *E. coli* is based on the high complexity of virulence factors and their combinations [23]. In the present study, 60 patterns of combinations were found among the 22 genes surveyed, indicating the high genotypic variability of *E. coli* isolates associated with clinical bovine mastitis, making it impossible to choose a characteristic virulence profile, although Guerra et al. [24] suggested *traT*, *ecpA* and *ompT* as a common feature among MPEC isolate genes. One B2 isolate exhibited 12 virulence genes among the 23 studied, showing great pathogenic potential, including genes for all groups of investigated virulence factors such as adhesins (*fimH* and *ecpA*), toxins (*hlyA, vat, cdt*), siderophores (*irp2, iucD, sitA*), invasins (*ibeA*) and serum resistance (*traT, KpsMTII* and *ompT*).

Through PFGE, we found that 11.8% of *E. coli* isolates were grouped into six clusters, among the 103 pulsotypes generated, again demonstrating the great genetic variability of environmental strains of *E. coli*. Moser et al. [25], using a 95% similarity coefficient, reported that 89.1% of bovine mastitis *E. coli* in their studies were genotypically different on the basis of PFGE analysis. We observed isolates belonging to the same cluster from the same quarter cow in different periods of lactation, showing persistence and adaptation in that environment. In addition, bacterial isolates from the same cluster were detected in different animals on the same farm, within three months, demonstrating a transmission through the environment.

Among the isolates that showed the AA pattern, five carried the *astA* gene and three carried the *shf* gene. As in the present study, Zhou et al. [26] reported the presence of the *astA* gene in *E. coli*, but none of them had EAEC virulence markers, even though they came from patients with gastroenteritis during an outbreak in Japan. This gene is widely distributed in a variety of *E. coli* pathotypes associated with intestinal diseases, as well as in *E. coli* isolates obtained from healthy subjects [16] and has been referred to as one of the three most common virulence factors associated with bovine mastitis [27]. Regarding the *shf* gene, this is the first report in cases of bovine mastitis, considering the literature reviewed, although participation of the protein encoded by this gene in the pathogenicity of MPEC isolates is still unknown.

The production of ESBL can vary considerably; in the present work, it was 4.5%, but results have ranged from 0.3% in France [28] to 75% in Germany [29]. Such a discrepancy may be related to the incidence of clinical bovine mastitis in different countries, the main reason for the frequent and prolonged use of antimicrobial agents, which exert selective pressure leading to the emergence and dissemination of resistant isolates [30].

Genes that encode temoniera enzymes (TEM), sulfhydryl variable (SHV), cefotaximase-Munich (CTX-M) and cephalomycinase (CMY) β-lactamases have proven to be more successful in dissemination and are predominant in Gram-negative bacteria [31], in addition to being commonly observed in multidrug-resistant *E. coli* cases [32]. Beta-lactamase CTX-M, especially CTX-M-15, has emerged as the most dominant type of ESBL in the world [33]. *E. coli*-producing CTX-M-15 has often been isolated from several sources, with production animals as well-established reservoirs, making it possible for transmission from animals to humans [27]. This fact was verified by Madec et al. [34], who reported that plasmids carrying the *bla*_CTX-M-15_ gene in *E. coli* isolated from cattle in ten different regions in France were highly similar to those found in ESBL-producing *E. coli* isolates from humans.

The *bla*_CTX-M-8_ gene had not been previously reported in cases of clinical mastitis in cows so far, although a Brazilian study identified it in dairy buffalo feces [35], highlighting a new reservoir of ESBL producers. In 2000, the *_bla_*_CTX-M-8_ gene was first described in *Enterobacteriaceae* resistant to cefotaxime isolated in Brazil [36]. Since then, it has been widely identified in *E. coli* isolates in South America (Uruguay, Argentina, and French Guiana), North America (USA and Canada), Africa (Kenya and Tunisia), Asia (Japan) and Europe (Spain, United Kingdom, and Germany), including strains isolated from poultry in the United Kingdom, Sweden, Japan and Tunisia, mainly that imported from South America [35], suggesting the possibility of transmission of isolates carrying *bla*_CTX-M-8_ through contaminated food, highlighting the potential of this gene to become endemic in the world.

The emergence of multidrug-resistant bacteria from animal and human origins has become a threat on a global level [37]. Bovine mastitis is a major disease in the dairy industry, and it is estimated that the treatment of mammary infections represents >80% of the use of antimicrobials on farms. In addition, more than 30 different drugs are commercially available for dry-off therapy or treatment during lactation [38]. The identification of resistance genes for different antimicrobials in *E. coli* strains isolated from cows sampled with clinical mammary infections agrees with similar studies [39], and could be attributed to the improper use of drugs in therapy during lactation or dry-cow therapy [5,40], including by *E. coli* infections [41]. In this regard, the culture of milk on farms (that has allowed the better decision of therapy during lactation) or other laboratories, selective dry-cow therapy (restricted to quarters infected over the lactation period) and identification of causal pathogens of mastitis to the decision-making of therapy, segregation or culling animals, are procedures related to the proper use of antimicrobials on dairy farms [5,40], which may decrease the selective pressure leading to multidrug-resistant bacteria.

A set of measures have been recommended for the management and control/prevention of bovine mastitis in dairy herds, involving milking procedures, housing, nutrition, genetics, diagnosis and therapeutic approaches [42]. Regarding milking, efforts for proper milking-machine operation, routine milking-equipment evaluations, adequate time for teat stimulation, use of gloves for milkers’ hands and individual towels for drying teats, a routine of cow drying prophylaxis and a lack of teat-end lesions have usually been recommended. Identification of mastitis cases include a routine of clinical/subclinical diagnosis and somatic cell counts, microbiological culture of milk (on farms or laboratories) and identification of pathogens. Therapy procedures involve treatment of clinical cases, selective dry cow therapy in addition to teat sealant and the segregation and culling of chronically infected animals. Environmental hygiene care in dairy herds includes adequate pre- and post-milking conditions, and clean and dry bedding. In addition, optimization of the immune response of cows (selenium and vitamin E supplementation), genetic selection for mastitis resistance and preventive measures for primigravid heifers should also be considered [5,40]. In particular, for the control/prevention of bovine mastitis-related environmental pathogens, e.g., *E. coli*, pre-milking antisepsis, clean and dry bedding, checking pre-and post-milking environment, dry cow therapy, vaccine prophylaxis, and offering feed immediately post-milking are conditions/procedures that should be considered. In parallel, advances in molecular biology for diagnosis, modern vaccines and novel therapeutic tools as alternatives to antibiotics are recent efforts aimed at providing new perspectives for the control/treatment of bovine mastitis [42].This study was limited by the absence of statistical analyses, which could not be performed because of the small sample size of certain phylogenetic groups (B2, C, D, E, F, E. clade I, and unknown), providing representativeness less than or equal to three isolates.

The ExPEC isolates showed great genetic heterogeneity, with no characteristic genetic pattern for the MPEC pathotype. In addition, this is the first report of the *papA, shf, ireA, sat* and *bla*_CTX-M-8_ genes in MPEC. As some isolates showed patterns of AD or AA, it is possible that some of them have diarrheagenic potential, despite not having the classic DEC markers. In addition, the presence of *E. coli* resistant to certain antimicrobial agents or carriers of resistance genes reinforces the concerns regarding the emergence of multidrug-resistant isolates present in the environment, animals and humans since *E. coli* is environmental and opportunistic, confirming the possibility of horizontal transfer of these resistance forms.

## Figures and Tables

**Table 1:** Distribution of extraintestinal *Escherichia coli* virulence genes among the phylogenetic groups, in 110 isolates obtained from the milk of cows with clinical mastitis.

Gene	Phylogenetic Groups								
	A (*n* = 56)	B1 (*n* = 42)	B2 (*n* = 1)	C (*n* = 2)	D (*n* = 3)	E (*n* = 1)	F (*n* = 1)	*E.* clade I (*n* = 1)	Unknown (*n* = 3)
*fimA*	11	13	0	0	3	1	1	1	1
*fimH*	53	39	1	2	2	1	1	1	3
*ecpA*	34	29	1	1	3	1	1	0	2
*papA*	0	1	0	0	0	0	0	0	0
*papC*	0	1	0	0	0	0	0	0	0
*hlyA*	0	1	1	0	0	0	0	0	0
*sat*	0	0	0	1	0	0	0	0	0
*vat*	0	0	1	0	0	0	0	0	0
*cdt*	7	3	1	0	0	0	0	0	0
*iroN*	0	3	0	0	1	0	0	0	0
*irp2*	17	16	1	0	2	1	0	0	1
*iucD*	1	0	1	0	1	0	1	0	0
*ireA*	0	2	0	0	0	0	0	0	0
*sitA*	9	16	1	0	2	1	0	0	0
*ibeA*	0	0	1	0	1	0	0	0	0
*traT*	42	35	1	0	2	1	0	1	3
*KpsMTII*	0	1	1	0	1	0	1	0	0
*ompT*	32	32	1	1	3	1	1	1	3

## Data Availability

The raw data of this study will be made available by the authors (corresponding author), without reservation, to any qualified researcher.

## Supplementary Materials

The following supporting information can be downloaded at: https://www.mdpi.com/article/10.3390/pathogens11121435/s1, Table S1: Primers and their characteristics used for the identification of genes in diarrheagenic *Escherichia coli* isolates obtained from milk of cows with clinical mastitis; Table S2: Primers and their characteristics used for the identification of genes in extraintestinal pathogenic *Escherichia coli* (ExPEC), isolates obtained from milk of cows with clinical mastitis; Table S3: Primers and their characteristics used for the identification of genes in enteroaggregative *Escherichia coli* (EAEC); Table S4: Primers and their characteristics used for the identification of antimicrobial resistance gene in *Escherichia coli* isolates obtained from milk of cows with clinical mastitis; Table S5: Distribution profile of virulence genes among 110 *Escherichia coli* isolates; Table S6: Susceptibility to antimicrobial agents in *E. coli* isolates, considering the phylogenetic group, isolated from milk of cows with subclinical mastitis; Figure S1: Dendrogram of PFGE patterns of 110 *E. coli* isolates obtained from milk of cows with clinical mastitis; Figure S2: Adherence patterns of *Escherichia coli* isolated from clinical mastitis on HeLa cells; (A) Aggregative adhesion; (B) Diffuse adhesion; (C) Non-characteristic adhesion; (D) Non-adherent. (1000× magnification) (C and D data not shown). References [43,44,45,46,47,48,49,50,51,52,53,54,55,56,57,58,59,60,61,62,63,64,65,66,67,68,69,70,71,72,73,74,75,76,77,78,79,80,81,82,83,84] are cited in the Supplementary Materials.
