# Supplementary material for: Genetic and Antimicrobial Resistance Profiles of Mammary Pathogenic E. coli (MPEC) Isolates from Bovine Clinical Mastitis
Source: Pathogens. 2022 Nov 28;11(12):1435. doi: 10.3390/pathogens11121435 (PMC9781227; doi:10.3390/pathogens11121435)
Supplement: Supplementary file 1 [file pathogens-11-01435-s001.zip › Supplementaries_Tables_1_to_6.pdf]

Supplementary Table S1. Primers and their characteristics used for the identification of genes in diarrheagenic *Escherichia coli* isolates obtained from milk of cows with clinical mastitis.

Gene	Sequence (5' → 3')	Annealing temperature (°C)	Amplicom (bp)	Reference
eae	F: CTGAACGGCGATTACGCGAA<br>R: CCAGACGATACGATCCAG	52	917	[46]
bfpA	F: AATGGTGCTTGCGCTTGCTGC<br>R: GCCGCTTTATCCAACCTGGTA	52	326	[43]
stx1	F: ATAAATCGCCATTCGTTGACTAC<br>R: AGAACGCCCACTGAGATCATC	52	180	[45]
stx2	F: GGCACTGTCTGAAACTGCTCC<br>R: TCGCCAGTTATCTGACATTCTG	49	255	[45]
ltA	F: GGCGACAGATTATACCGTGC<br>R: CCGAATTCTGTTATATATGTC	50	322	[49]
est	F: ATTTTTMTTTCTGTATTRTCTT<br>R: CACCCGGTACARGCAGGATT	52	190	[43]
ipaH	F: GTTCCTTGACCGCCTTTCCGATACCGTC<br>R: GCCGGTCAGCCACCCTCTGAGAGTAC	52	619	[50]
aggR	F: GTATACACAAAAGAAGGAAGC<br>R: ACAGAATCGTCAGCATCAGC	52	254	[50]
aatA	F: CTGGCGAAAGACTGTATCAT<br>R: CAATGTATAGAAATCCGCTGTT	55	630	[47]
daaE	F: CACTGTGGGCTCCGCGCAAGC<br>R: CGGTGAGGTTCAGTGTGTAT	55	419	[44]
ehxA	F: GGTGCAGCAGAAAAAGTTGTAG<br>R: TCTCGCCTGATAGTGTTTGGTA	57	1551	[48]

bp: base pair; (°C): centigrade degrees

EPEC-E2348/69 (*eae*$^+$, *bfpA*$^+$), EHEC-EDL933 (*stx1*$^+$, *stx2*$^+$, *ehxA*$^+$), ETEC-H10407 (*elt*$^+$, *est*$^+$), EIEC-EDL1284 (*ipaH*$^+$), EAEC-042 (*aatA*$^+$, *aggR*$^+$), STEC-C1845 (*daaE*$^+$) were used as positive controls and *E. coli* C600, as negative control in PCR tests.

Supplementary Table S2. Primers and their characteristics used for the identification of genes in extraintestinal pathogenic *Escherichia coli* (ExPEC), isolates obtained from milk of cows with clinical mastitis.

Gene	Sequence (5’ → 3’)	Annealing temperature (°C)	Amplicom (bp)	Reference
Adhesins				
sfaDE	F: CGGAGGAGTAATTACAAACCTGGCA<br>R: CTCCGGAGAACTGGGTGCATCTTAC	65	410	[58]
fimA	F: AGTTAGGACAGGTTCGTACCGCAT<br>R: AAATAACGCGCCTGGAACGGAATG	60	331	[55]
fimH	F: TGCAGAACGGATAAGCCGTGG<br>R: GCAGTCACCTGCCCTCCGGTA	53	508	[57]
iha	F: CAGGTCGGGGTTACCAAGT<br>R: CAAATGGCTCTCTTCCGTCAATGC	50	925	[60]
ecpA	F: GCAACAGCCAAAAAAGACACC<br>R: CCAGGTCGCGTCGAACT	50	477	[55]
papA	F: ATGGCAGTGGTGTCTTTTGGTG<br>R: CGTCCCACCATACGTGCTCTTC	65	720	[57]
papC	F: GACGGCTGTACTGCAGGGTGTGGCG<br>R: ATATCCTTTCTGCAGGGATGCAATA	65	328	[58]
afaBC III	F: GCTGGGCAGCAAACTGATAACTCTC<br>R: CATCAAGCTGTTTGTTCGTCCGCCG	65	750	[58]
Toxins				
hlyA	F: AACAAGGATAAGCACTGTTCTGGCT<br>R: ACCATATAAGCGGTCATTCCCGTCA	60	1177	[57]
cnf1	F: AGATGGAGTTTCCTATGCAGGAG<br>R: CATTCAGAGTCCTGCCCTCATTATT	53	498	[62]
sat	F: TGCTGGCTCTGGAGGAAC<br>R: TTGAACATTCAGAGTACCGGG	52	667	[54]
vat	F: AACGGTTGGTGGCAACAATCC<br>R: AGCCCTGTAGAATGGCGAGTA	52	420	[51]
cdt	F: GAAARTAAATGGAAYAYAMATGTCC<br>R: AATCWCCWRSAATCATCCAGTTA	45	466	[61]

Supplementary Table S2. Primers and their characteristics used for the identification of genes in Extraintestinal Pathogenic *Escherichia coli* (ExPEC), isolates obtained from milk of cows with clinical mastitis. (continuation)

Gene	Sequence (5’ → 3’)	Annealing temperature (°C)	Amplicom (bp)	Reference
Siderophores				
iroN	F: AAGTCAAAGCAGGGGTTGCCCG<br>R: GACGCCGACATTAAGACGCAG	65	665	[56]
irp2	F: AAGGATTCGCTGTTACCGGAC<br>R: TCGTCGGGCAGCGTTTCTTCT	57	264	[52]
iucD	F: AAGTGTCGATTTTATTGGTGTA<br>R: CCATCCGATGTCAGTTTTCTG	50	760	[53]
ireA	F: ATTGCCGTGATGTGTTCTGC<br>R: CACGGATCACTTCAATGCGT	45	384	[54]
sitA	F: AGGGGGCACAACTGATTCTCG<br>R: TACCGGGCCGTTTTCTGTGC	54	608	[59]
Invasins				
ibe10	F: AGGCAGGTGTGCGCCGCGTAC<br>R: TGGTGCTCCGGCAAACCATGC	63	170	[57]
Serum resistance				
traT	F: GGTGTGGTGCGATGAGCACAG<br>R: CACGGTTCAGCCATCCCTGAG	58	290	[57]
KpsMTII	F: GCGCATTTGCTGATACTGTTG<br>R: CATCCAGACGATAAGCATGAGCA	63	272	[57]
ompT	F: ATCTAGCCGAAGAAGGAGGC<br>R: CCCGGGTCATAGTGTTCATC	59	559	[56]

bp: base pair; (°C): centigrade degrees

Strains *E. coli* 042 (*irp2*$^+$), EDL933 (*iha*$^+$), C1845 (*sat*$^+$), RS218 (*sfaDE*$^+$, *vat*$^+$, *iroN*$^+$, *ibe10*$^+$, *ompT*$^+$, *traT*$^+$), J96 (*fimA*$^+$, *fimH*$^+$, *papA*$^+$, *papC*$^+$, *hlyA*$^+$, *cnf1*$^+$, *KpsMTII*$^+$), A30 (*ecpA*$^+$, *afaBC*$^+$), V27 (*ireA*$^+$), 1551-2 (*cdt*$^+$), EC40 (*sitA*$^+$) e *Shigella flexneri* SA101 (*iucD*$^+$) were used as positive controls and *E. coli* C600 (*afaBC*$^-$, *traT*$^-$, *cdt*$^-$, *astA*$^-$), J53 (*sfaDE*$^-$, *iha*$^-$, *papA*$^-$, *papC*$^-$, *hlyA*$^-$, *cnf1*$^-$, *sat*$^-$, *vat*$^-$, *irp2*$^-$, *iucD*$^-$, *ireA*$^-$, *sitA*$^-$, *ibe10*$^-$, *Kps*MTII$^-$), A30 (*iroN*$^-$), 042 (*ompT*$^-$), *Klebsiella pneumoniae* CF504 (*fimA*$^-$, *fimH*$^-$) and *Shigella flexneri* SA101 (*ecpA*$^-$) were negative controls in PCR tests.

Supplementary Table S3. Primers and their characteristics used for the identification of genes in enteroaggregative *Escherichia coli* (EAEC)

Gene	Sequence (5’ → 3’)	Annealing temperature (°C)	Amplicom (bp)	Reference
astA	F: CCATCAACACAGTATATCCGA<br>R: GGTCGCGAGTGACGGCTTTG	57	111	[74]
Adhesins				
aggA	F: CGCTTAGAAAGACCTCCAATA<br>R: GCCGGATCCTTAAAAATTAATTCCGGC	52	432	[73]
aafA	F: ACATGCATGCAAAAAATCAGAATGTTTGTT<br>R: CGGGATCCATTTGTCACAAGCTCAGC	65	550	[66]
agg3A	F: GTATCATTGCGAGTCTGGTATTCAG<br>R: GGGCTGTTATAGAGTAACTTCCAG	55	462	[64]
agg4A	F: TCCATTATGTCAGGCTGCAA<br>R: GGCGTTAACGTCTGATTTCC	55	411	[65]
agg5A	F: CATGTTCATTATCTATTAGTTCGCC<br>R: TCCACCGTACGTCGTCATTA	55	215	[70]
pilS	F: ATGAGCGTCATAACCTGTTC<br>R: CTGTTGGTTTCAGTTTGAT	50	533	[67]
Toxins				
pic	F: GGGTATTGTCCGTTCCGAT<br>R: ACAACGATACCGTCTCCCG	50	1175	[52]
sigA	F: CCGACTTCTCACTTTCTCCCG<br>R: CCATCCAGCTGCATAGTGTTTG	55	430	[51]
sepA	F: GCAGTGGAAATATGATGCGGC<br>R: TTGTTCAGATCGGAGAAGAACG	55	794	[72]
pet	F: GTGTTTCAACCAGGTTCAACA<br>R: CCTTCACCAATTTTATGCAGT	50	1037	[69]

Supplementary Table S3. Primers and their characteristics used for the identification of genes in enteroaggregative *Escherichia coli* (EAEC). (continuation)

Gene	Sequence (5' → 3')	Annealing temperature (°C)	Amplicom (bp)	Reference
Miscellany				
shf	F: ACTTTCTCCCGAGACATTC<br>R: CTTTAGCGGGAGCATTCAT	55	613	[52]
aap	F: CTTTTCTGGCATCTTGGGT<br>R: GTAACAACCCCTTTGGAAGT	55	232	[52]
aaiA	F: CCCACGAGTACAGATAACG<br>R: GTTTTCAGGATTGCCATTAG	50	476	[68]
aaiC	F: ATTGTCCTCAGGCATTTCACACG<br>R: GACACCCCTGATAAACAAC	55	215	[71]
aaiG	F: GGGAGTGTTTCAGTCTGGAC<br>R: TTATCGGTGATAAGACTACCACTT	55	785	[63]

bp: base pair; (°C): centigrade degrees

Strains *E. coli* 042 (*astA*$^+$, *aafA*$^+$, *pic*$^+$, *pet*$^+$, *aap*$^+$, *aaiA*$^+$, *aaiC*$^+$, *aaiG*$^+$, *shf*$^+$), 17-2 (*aagA*$^+$), RNT785 (*agg3A*$^+$), BA1116 (*agg4A*$^+$), 67330 (*agg5A*$^+$), C1096 (*pilS*$^+$) and *Shigella flexneri* SA101 (*sigA*$^+$, *sepA*$^+$) were used as positive controls and *E. coli* C600 was used as negative control in PCR tests.

Supplementary Table S4. Primers and their characteristics used for the identification of antimicrobial resistance gene in *Escherichia coli* isolates obtained from milk of cows with clinical mastitis.

Gene	Sequence (5' → 3')	Annealing temperature (°C)	Amplicom (bp)	Reference
bla_{TEM}	F: ATGAGTATTCAACATTTCCGTG<br>R: TTACCAATGCTTAATCAGTGAG	55	840	[78]
bla_{SHV}	F: ATTTGTCGCTTCTTTACTCGC<br>R: TTTATGGCGTTACCTTTGACC	60	1.051	[84]
$bla_{\mathrm{CTX\text{-}M}}$	F: TTTGCGATGTGCAGTACCAGTAA<br>R: CGATATCGTTGGTGGTGCCATA	51	544	[75]
$bla_{\mathrm{CTX\text{-}M\text{-}2}}$	F: AAATGTGCTGCTCCTTTCGTGAGC<br>R: AGGGTTCGTTGCAAGACAAGACTG	60	1.112	[77]
$bla_{\mathrm{CTX\text{-}M\text{-}8}}$	F: GCAAGCGCATTTTTTGTTTTT<br>R: GACGACTTTCTGCCTTCTGC	55	307	[76]
$bla_{\mathrm{CTX\text{-}M\text{-}15}}$	F: CACACGTGGAATTTAGGGACT<br>R: GCCGTCTAAGGCGATAAACA	51	996	[82]
$bla_{CMY\text{-}2}$	F: ATGATGAAAAAATCGTTATGC<br>R: TTGCAGCTTTTCAAGAATGCGC	55	1143	[83]
bla_{KPC}	F: CGTCTAGTTCTGCTGTCTTG<br>R: CTTGTCATCCTTGTTAGGCG	54	798	[81]
bla_{NDM}	F: GGTTTGGCGATCTGGTTTTC<br>R: CGGAATGGCTCATCACGATC	54	621	[81]
mcr-1	F: GGTCAGTCCGTTTGTTC<br>R: CTTGGTCGGTCTGTA	60	309	[80]
mcr-2	F: ATGACATCACATCACTCTTGG<br>R: TTACTGGATAAATGCCGCGC	52	715	[79]

bp: base pair; (°C): centigrade degrees

Sequenced *E. coli, Klebsiella pneumoniae, K. oxytoca* and *Pseudomonas aeruginosa* strains of our Laboratory Collection were used as positive controls. Ultrapure water was used as negative control.

Supplementary Table S5. Distribution profile of virulence genes among 110 *Escherichia coli* isolates

Profile of virulence genes	Nº of isolates
fimH	1
fimH, ecpA	2
ecpA, irp2	1
fimH, irp2	1
fimH, traT	5
fimH, ecpA, irp2	1
fimH, ecpA, traT	6
fimH, ecpA, sitA	1
fimH, ecpA, irp2	1
fimH, ecpA, astA	1
fimH, traT, ompT	16
fimA, fimH, traT	3
fimH, ecpA, ompT	1
fimA, fimH, ecpA	1
fimH, irp2, traT, ompT	2
fimH, ecpA, irp2, astA	1
fimA, ecpA, sitA, traT	2
fimH, ecpA, sat, ompT	1
fimH, ecpA, sitA, ompT	1
ecpA, irp2, sitA, ompT	1
fimH, ecpA, irp2, traT	1
fimA, fimH, irp2, astA	1
fimH, irp2, traT, ompT	2
fimA, fimH, ecpA, traT	1
fimH, ecpA, traT, ompT	7
fimA, fimH, traT, ompT	3
fimH, irp2, sitA, traT	1
fimA, fimH, ecpA, traT, ompT	3

Supplementary Table S5. Distribution profile of virulence genes among 110 *Escherichia coli* isolates (continuation)

Profile of virulence genes	Nº of isolates
fimA, fimH, ecpA, traT, astA	1
fimH, ecpA, sitA, ompT, cdt	2
ecpA, irp2, sitA, ompT, cdt	1
fimH, ecpA, irp2, ompT, cdt	1
fimH, ecpA, irp2, traT, ompT	2
fimH, ecpA, traT, ompT, cdt	2
fimH, ecpA, irp2, sitA, ompT	1
fimA, fimH, ecpA, irp2, sitA	1
fimA, fimH, ecpA, irp2, traT	1
fimH, ecpA, traT, ompT, astA	2
fimH, irp2, sitA, traT, ompT	1
fimA, fimH, ecpA, sitA, ompT	1
fimH, ecpA, sitA, traT, ompT	2
fimH, papA, papC, traT, ompT	1
iroN, irp2, ireA, sitA, traT, ompT	1
fimA, fimH, ecpA, traT, ompT, KpsMTII	1
fimA, fimH, ecpA, irp2, iucD, traT	1
fimH, ecpA, irp2, sitA, traT, ompT	2
fimH, ecpA, irp2, traT, ompT, cdt	1
fimH, ecpA, sitA, traT, ompT, cdt	1
fimA, ecpA, sitA, traT, ompT, cdt	1
fimA, ecpA, irp2, sitA, ibe10, ompT	1
fimA, fimH, ecpA, sitA, traT, ompT	2
fimA, fimH, ecpA, irp2, traT, ompT	4
fimA, fimH, ecpA, iucD, ompT, KpsMTII	1
fimH, ecpA, irp2, sitA, traT, ompT, astA	1
fimA, fimH, ecpA, irp2, sitA, traT, ompT	1
fimA, fimH, ecpA, irp2, traT, ompT, astA	1
ecpA, iroN, irp2, ireA, sitA, traT, ompT	1
fimA, fimH, ecpA, iroN, irp2, iucD, sitA, traT, ompT, KpsMTII	1
fimA, fimH, ecpA, hlyA, iroN, irp2, sitA, traT, ompT, cdt	1
fimH, ecpA, hlyA, vat, irp2, iucD, sitA, ibe10, traT, ompT, cdt, KpsMTII	1

Supplementary Table S6. Susceptibility to antimicrobial agents in *E. coli* isolates, considering the phylogenetic group, isolated from milk of cows with subclinical mastitis.

Antimicrobial Agents		Phylogenetic Groups									Total	
		A (n = 56)	B1 (n = 42)	B2 (n = 1)	C (n = 2)	D (n = 3)	E (n = 1)	F (n = 1)	*E.* clade I (n = 1)	Unknown (n = 3)	No. isolates	%
Ampicillin	S	50	34	1	2	3	1	1	1	3	96	87.3
	I	0	0	0	0	0	0	0	0	0	0	0
	R	6	8	0	0	0	0	0	0	0	14	12.7
Cefepime	S	53	37	1	2	3	1	1	1	3	102	92.7
	I	1	1	0	0	0	0	0	0	0	2	1.8
	R	2	4	0	0	0	0	0	0	0	6	5.5
Cefotaxime	S	51	38	1	2	3	1	1	1	3	101	91.8
	I	1	1	0	0	0	0	0	0	0	2	1.8
	R	4	3	0	0	0	0	0	0	0	7	6.4
Ceftriaxone	S	52	39	1	2	3	1	1	1	3	103	93.6
	I	0	0	0	0	0	0	0	0	0	0	0
	R	4	3	0	0	0	0	0	0	0	7	6.4
Cefoxitin	S	55	42	1	2	3	1	1	1	3	109	99.1
	I	1	0	0	0	0	0	0	0	0	1	0.9
	R	0	0	0	0	0	0	0	0	0	0	0
Ceftazidime	S	51	41	1	2	3	1	1	1	2	103	93.6
	I	1	0	0	0	0	0	0	0	0	1	0.9
	R	4	1	0	0	0	0	0	0	1	6	5.5
Ceftiofur	S	54	38	1	2	3	1	1	1	3	104	94.5
	I	0	0	0	0	0	0	0	0	0	0	0
	R	2	4	0	0	0	0	0	0	0	6	5.5
Aztreonam	S	52	40	1	2	3	1	1	1	2	103	93.6
	I	1	1	0	0	0	0	0	0	0	2	1.8
	R	3	1	0	0	0	0	0	0	1	5	4.5
Gentamicin	S	53	41	1	2	3	1	1	1	3	106	96.4

	I	0	1	0	0	0	0	0	0	0	1	0.9
	R	3	0	0	0	0	0	0	0	0	3	2,7
	S	48	30	0	2	2	1	1	0	3	87	79,1
Streptomycin	I	5	8	0	0	1	0	0	0	0	16	14,5
	R	3	4	1	0	0	0	0	1	0	7	6,4
	S	47	33	1	2	3	1	1	0	1	89	80,9
Tetracycline	I	0	0	0	0	0	0	0	0	0	0	0
	R	9	9	0	0	0	0	0	1	2	21	19,1

S: Susceptible; I: Intermediate; R: Resistant
