# Supplementary material for: Genetic and Antimicrobial Resistance Profiles of Mammary Pathogenic E. coli (MPEC) Isolates from Bovine Clinical Mastitis
Source: Pathogens. 2022 Nov 28;11(12):1435. doi: 10.3390/pathogens11121435 (PMC9781227; doi:10.3390/pathogens11121435)
Supplement: Supplementary file 1 [file pathogens-11-01435-s001.zip › Supplementary_Figure S1.pdf]

Supplementary Figure S1. Dendrogram of PFGE patterns of 110 *E. coli* isolates obtained from milk of cows with clinical mastitis

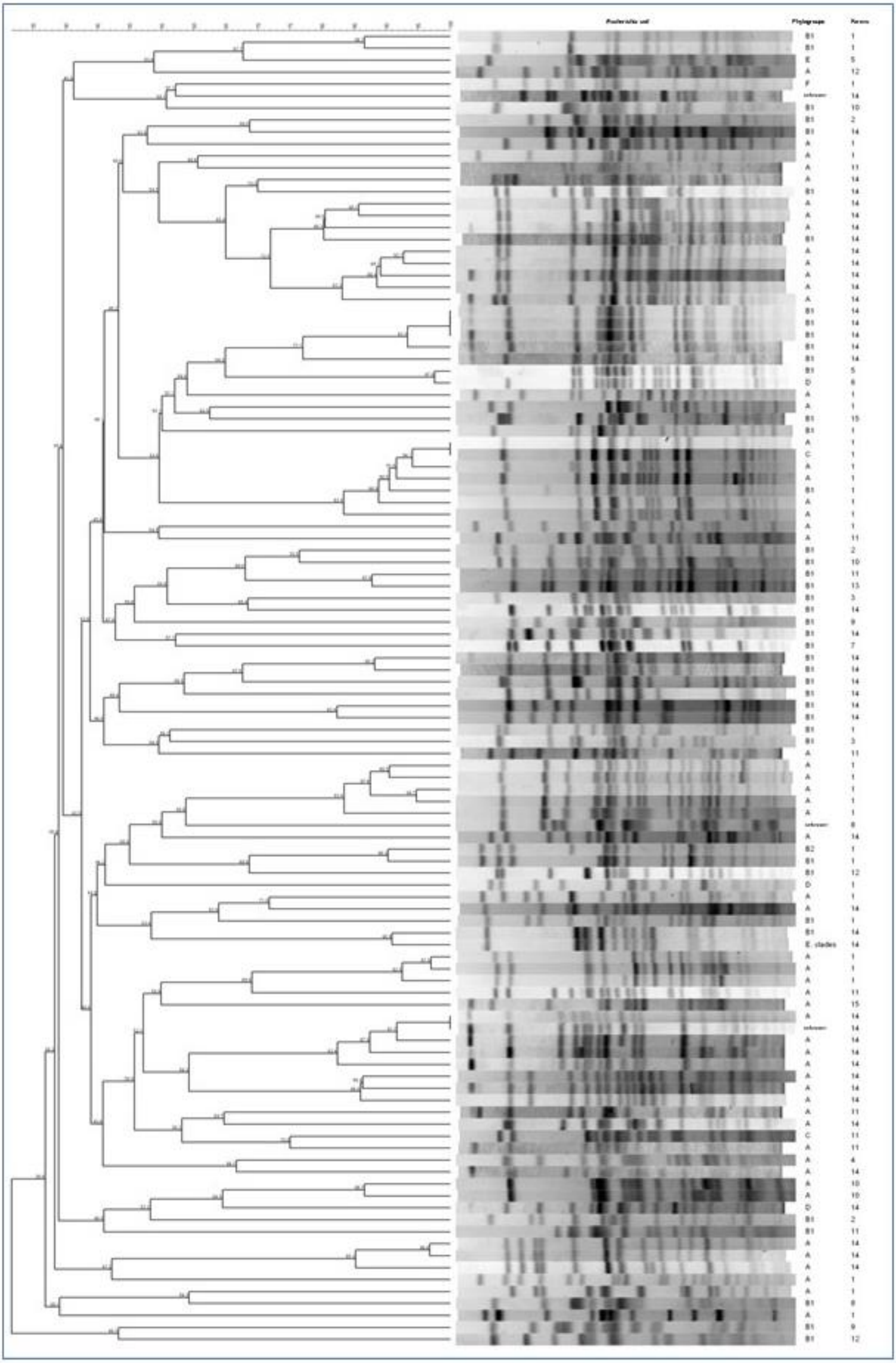
E. clades
