# Supplementary material for: Genetic and Antimicrobial Resistance Profiles of Mammary Pathogenic E. coli (MPEC) Isolates from Bovine Clinical Mastitis
Source: Pathogens. 2022 Nov 28;11(12):1435. doi: 10.3390/pathogens11121435 (PMC9781227; doi:10.3390/pathogens11121435)
Supplement: Supplementary file 1 [file pathogens-11-01435-s001.zip › Supplementary_Figure S2.pdf]

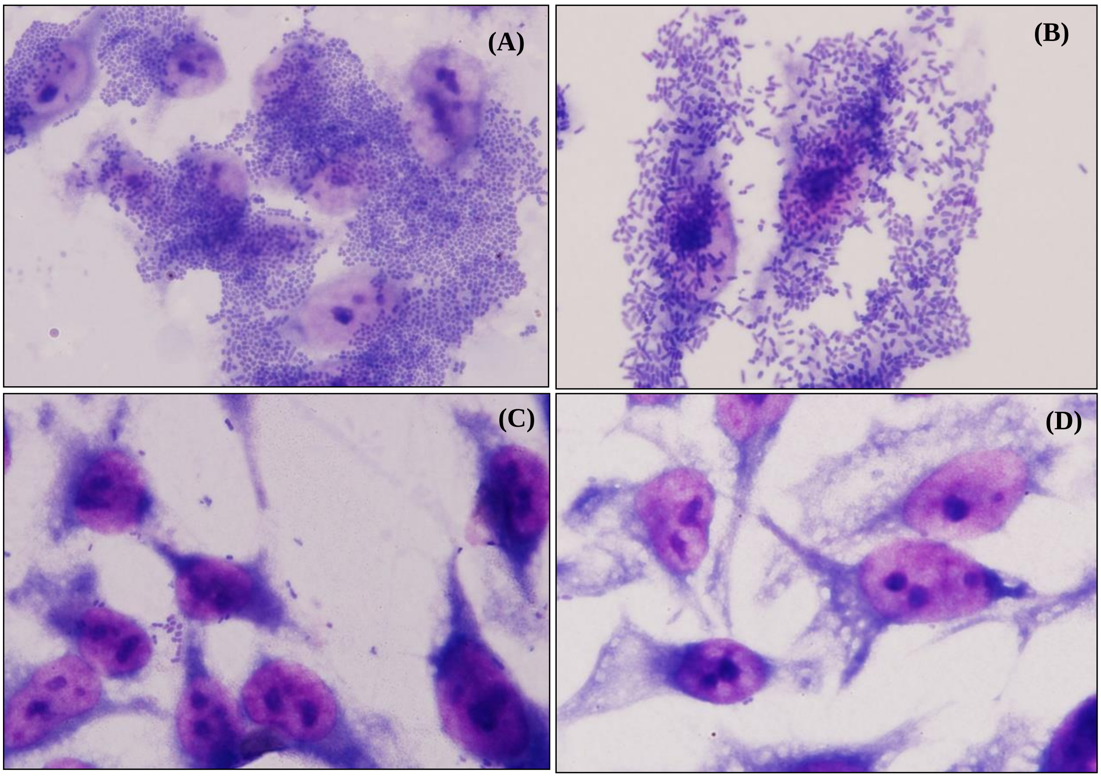

Supplementary Figure S2. Adherence patterns of *Escherichia coli* isolated from clinical mastitis on HeLa cells. (A) Aggregative adhesion. (B) Diffuse adhesion. (C) Non-characteristic adhesion. (D) Non-adherent. (1000x magnification) (C and D data not shown).
